# Antioxidant Efficacy of Litchi (*Litchi chinensis* Sonn.) Pericarp Extract in Sheep Meat Nuggets

**DOI:** 10.3390/antiox5020016

**Published:** 2016-05-18

**Authors:** Arun K. Das, Vincent Rajkumar, Pramod K. Nanda, Pranav Chauhan, Soubhagya R. Pradhan, Subhasish Biswas

**Affiliations:** 1 ICAR-Indian Veterinary Research Institute, Eastern Regional Station, Kolkata 700 037, India; npk700@gmail.com (P.K.N.); dr.pranav.chauhan@gmail.com (P.C.); 2 ICAR-Central Institute for Research on Goats, Farah, Mathura 281 122, India; vrvet@rediffmail.com; 3 Department of Livestock Products Technology, West Bengal University of Animal and Fishery Sciences, Kolkata 700 037, India; soubhagyaovc@gmail.com (S.R.P.); lptsubhasish@gmail.com (S.B.)

**Keywords:** antioxidant, lipid oxidation, litchi fruit, meat nuggets, pericarp extract, total phenolics

## Abstract

In the present study, the efficacy of litchi fruit pericarp (LFP) extract (0.5%, 1.0% and 1.5% concentration) in retarding lipid oxidation of cooked sheep meat nuggets was evaluated and compared to butylated hydroxyl toluene (BHT, 100 ppm). The total phenolic content and antioxidant potential of LFP extracts were determined. The thiobarbituric acid reactive substance (TBARS) values were evaluated to assess the potential of LFP extracts as natural antioxidants for oxidative stability of cooked nuggets during 12 days of refrigerated storage. Results show that total phenolics content in 10 mg LFP powder was comparable to 100 ppm BHT, but 15 mg LFP powder had significantly higher (*p* < 0.05) total phenolics content and reducing power than the synthetic antioxidant. LFP extract did not affect pH, cooking yield and the sensory attributes of cooked nuggets. Non-treated control and nuggets with 1.0% LFP extract had significantly lower total phenolics than nuggets with 1.5% extract and BHT. TBARS values were significantly lower (*p* < 0.05) throughout the storage period in cooked meat nuggets containing either LFP extract or BHT than in non-treated control. Results indicate that LFP extracts are promising sources of natural antioxidants and can potentially be used as functional food additives in meat products at 1.5% without affecting products’ acceptability.

## 1. Introduction

Lipid oxidation and auto-oxidation play a major role in quality deterioration, reduced shelf-life and a decline in the nutritive value of muscle foods. Oxidation can also cause other detrimental effects, such as loss of essential fatty acids, flavor and discoloration, leading to changes in organoleptic attributes [1] and the formation of genotoxic and cytotoxic compounds [2]. Lipid oxidation can be influenced by several factors, such as the degree of lipid unsaturation, muscle type, animal diet and the additives used during processing, cooking and storage. Additionally, reduced pH of muscle food enhances lipid oxidation because of intensified autoxidation of hemoglobin [3]. To control quality deterioration in muscle foods due to lipid oxidation, synthetic antioxidants, such as butylated hydroxytoluene (BHT) and butylated hydroxy anisole (BHA), are extensively used [4,5].

Recently, the use of synthetic antioxidants has been viewed negatively due to toxicity, adverse effects on human health and food safety [6]. Therefore, research on safe and effective natural antioxidants from natural sources, such as phenolic compounds, is being explored to control lipid oxidation. Presently, natural antioxidants present in foods and other biological materials have attracted considerable attention because of their safety and potential nutritional and therapeutic values [7,8]. Phenolic extract from plants, as well as many phenolic compounds have been successfully demonstrated as natural antioxidants in retarding lipid oxidation in different foods [9]. The antioxidant effects of various plant extracts, as well as individual phenolic compounds in different muscle food systems have been evaluated and tested by various workers [1,5,7,10,11,12].

Litchi (*Litchi chinensis* Sonn.) is a tropical and subtropical fruit native to South East Asian countries and now widely cultivated throughout the world [13]. India is the second largest producer of litchi in the world after China. Litchi or lychee, a fruit with a rough brown pericarp surrounding a white flesh (aril), is popular for its delicious taste and possible health benefits [14]. Litchi fruit pericarp (LFP), which accounts for approximately 15% by weight of the whole fresh fruit, is usually discarded as waste during processing. Recent studies have reported that litchi pericarp contains significant amounts of polyphenols, flavonoids, anthocyanins and polysaccharides [15,16]. The major phenolics in LFP tissues were identified as epicatechin, procyanidin B4 and procyanidin B2 [13]. These phenolics from LFP have good antioxidant activity, anti-inflammatory, anti-carcinogenic and immune-modulatory properties [14,17,18]. However, to our knowledge, no studies have been conducted regarding the antioxidant potential of LFP extract in muscle foods. Herein, we report the first study of the use of LFP extract in a meat product formulation. The objectives of this study were to investigate the antioxidant potential of LFP, an abundant and under-utilized natural resource, and to evaluate its effectiveness in retarding lipid oxidation of cooked meat products.

## 2. Materials and Methods

### 2.1. Preparation of Litchi Fruit Pericarp Extract

Fresh and mature litchi fruit was purchased from the local market. The rough brown outer covers (pericarps) were carefully removed from fruits, cleaned with water and dried in an oven at 50 °C. After drying, a fine powder of pericarp was made using a home grinder. Ten grams of litchi pericarp powder were added in 100 mL boiled distilled water and left for 1 h followed by filtration through Whatman No. 1 filter paper to obtain a water extract of litchi fruit pericarp.

### 2.2. Sheep Meat Nugget Preparation

Sheep meat nuggets were prepared as per the procedure outlined by Das *et al.* [19]. Briefly, sheep meat from leg and loin cuts was collected from the experimental slaughterhouse and kept under frozen storage at −18 °C until further processing. Before processing, meat was thawed and cut into small cubes and minced (Tallers Ramon Model P-22, Barcelona, Spain). For meat emulsion preparation, salt, sugar, phosphate and nitrite were thoroughly mixed to the pre-weighed quantity of minced sheep meat in a bowl chopper (Seydelmann K20 Ras, Stuttgart, Germany), and ice flakes were added during chopping to maintain a lower temperature (8 ± 2 °C). Condiments, dry spice mix, fine wheat flour and LFP extract (0%, 1% and 1.5%) were added, and chopping was continued until uniform mixing of all ingredients. About 500 g of emulsion were placed in a mold and steam cooked for 40 min to prepare cooked meat blocks. Blocks were sliced and cut into small nuggets. Sheep meat nuggets were aerobically packed in low-density polyethylene (LDPE) pouches and kept at refrigerated temperature (4 ± 1 °C) for further analysis. The formulation for control and treated nuggets with LFP extracts and BHT (100 ppm) is present in Table 1. The whole experiment was replicated thrice.

### 2.3. Analysis of Litchi Fruit Pericarp Extract

#### 2.3.1. Analysis of Total Phenolics Content

Total phenolics content in litchi fruit pericarp extract and BHT were determined by the Folin-Ciocalteu (F-C) method [20]. Zero-point-seven-five microliters (0.75 µL) of Folin-Ciocalteu reagent were added in 100 µL of different dilutions of extract, and the final volume was made ten times with distilled water (7.65 mL). After 5 min, 0.75 mL of a sodium carbonate solution (7.5%) were added to each tube. The tubes were incubated for 90 min at room temperature in the dark, and absorbance (U-28000 Spectrophotometer, Hitachi, Tokyo, Japan) was measured against a blank at 725 nm. A standard curve was plotted using different concentrations of gallic acid, and the amount of total phenolics was calculated as gallic acid equivalents (GAE) in mg/g of dried LFP powder.

Total phenolics content in cooked sheep meat nuggets from control, BHT and extract-incorporated formulations was analyzed by using the Folin-Ciocalteu assay [21] with slight modifications. Briefly, five grams of nugget were homogenized with 25 mL of 70% acetone and kept overnight for extraction at refrigeration temperature. Suitable aliquots of extracts were taken in a test tube, and the volume was made to 0.5 mL with distilled water followed by the addition of 0.25 mL F-C (1 N) reagent and 1.25 mL sodium carbonate solution (20%). The tubes were vortex mixed, and the absorbance was recorded at 725 nm after 40 min.

#### 2.3.2. Radical Scavenging Activity Using the DPPH Assay

The DPPH (2,2-diphenyl-1-picrylhydrazyl) assay was performed according to the method of Fargere *et al.* [22]. An aliquot of the various concentrations of the LFP extract and BHT was mixed with 3 mL of DPPH in methanol (final concentration of 250 μM), and the mixture was vortexed vigorously. Tubes were then incubated at room temperature for 30 min in the dark, and the absorbance was taken at 517 nm. Radical scavenging activity (RSA) was calculated by the following equation: RSA% = (Absorbance_Control_ − Absorbance_Sample_/Absorbance_Control_) × 100.

#### 2.3.3. Ferric Reducing Antioxidant Power Assay

The ferric reducing antioxidant power of BHT and LFP extracts was determined according to the method of Oyaizu [23]. Different concentrations of the LFP extracts and 100 ppm BHT were mixed with 2.5 mL of phosphate buffer (0.2 M, pH 6.6) and 2.5 mL of 1% (*w*/*v*) potassium ferricyanide. Mixtures were incubated for 20 min at 50 °C followed by the addition of 2.5 mL of 10% trichloroacetic acid and then centrifuged at 700× *g* for 10 min. The supernatant (2.5 mL) was mixed with 2.5 mL of distilled water and 0.5 mL of ferric chloride (0.1% *w*/*v*). The absorbance was measured at 700 nm (U-28000 spectrophotometer, Hitachi, Japan). An increase in the absorbance of the reaction mixture indicated the reducing power of the sample.

### 2.4. Analysis of Cooked Sheep Meat Nuggets

#### 2.4.1. pH and Cooking Yield of Nuggets

The pH of the cooked nuggets was determined by blending 10 g of sample with 50 mL of distilled water for a minute in a homogenizer (model PT-MR-2100, Kinematica AG, Luzern, Switzerland). The pH values were measured using a standardized electrode attached to a digital pH meter (Systronics, Ahmedabad, India). The cooking yield of nuggets was determined by recording the weight of each meat block before and after cooking. The yield was calculated and expressed in percentage as: weight of cooked meat block/weight of raw meat block ×100.

#### 2.4.2. Sensory Evaluation of Sheep Meat Nuggets

A 10-member experienced panel evaluated sheep meat nuggets using an 8-point descriptive scale, where 8 denoted extremely desirable and 1 denoted extremely poor. Panelists were provided information about the nature of the experiments without disclosing the identity of samples and were asked to evaluate the samples for appearance, flavor, juiciness, texture and overall acceptability. Samples were warmed using a microwave oven for 1 min and served randomly to the panelists. The panelists were provided filtered water to rinse their mouth between samples

#### 2.4.3. Lipid Peroxidation of Nuggets during Storage

Lipid peroxidation of nuggets was recorded by measuring thiobarbituric acid-reactive substances (TBARS) at an interval of 3 days during refrigerated storage. The TBARS number (mg malonaldehyde/kg) of nuggets was estimated using the extraction method outlined by Witte *et al.* [24] with slight modifications, as the slurry was centrifuged at 3000× *g* for 10 min (Biofuge Primo R, Heraeus, Hanau, Germany) instead of filtration through Whatman No. 42.

### 2.5. Statistical Analysis

This study was replicated thrice, and in each replication, measurements of all parameters were done in duplicate. One-way ANOVA was conducted using SPSS software (Version 20.0, IBM Corp, Armonk, NY, USA) for the calculation of different mean values (pH, cooking yield, total phenolics, DPPH and sensory attributes), whereas lipid peroxidation was analyzed using two-way ANOVA with treatment and storage time as the main effects. Statistical significance was identified at the 95% confidence level (*p* < 0.05).

## 3. Results and Discussion

### 3.1. Total Phenolics Content of Litchi Pericarp Extract

**Figure 1:**
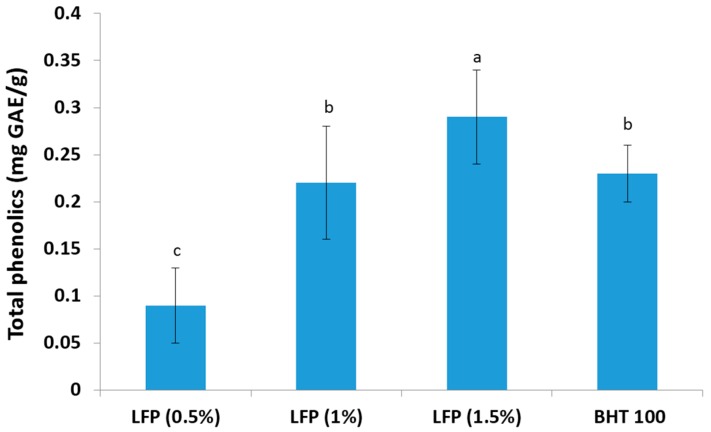
Total phenolics content in litchi fruit pericarp extracts (0.5%, 1.0% and 1.5%) and BHT (100 ppm). Mean values bearing different superscripts (a, b and c) differ significantly (*p* < 0.05).

It is well known that plant phenolic compounds, also called polyphenols, are a large and diverse class of compounds with one or more aromatic rings bearing hydroxyl substituent(s) and have an antioxidant potential due to their possibility to act as radical scavengers or free radical terminators. Antioxidant activity is significantly correlated with phenolic and flavonoid contents [25]. Therefore, the determination of total phenolics content is one of the most important parameters to estimate the amount of antioxidants in the plant materials. Total phenolics content of different concentrations of litchi fruit pericarp extract and BHT (100 pm) is presented in Figure 1. The results indicate that total phenolics content in 10 mg litchi fruit pericarp powder was comparable with 100 ppm BHT, but 15 mg pericarp powder had significantly higher (*p* < 0.05) total phenolics content than 100 ppm BHT. This study shows that litchi fruit pericarp powder is a good source of phenolic compounds containing about 18.36 mg (12.42 to 27.53) GAE/g dry weight. Li *et al.* [17] reported that litchi fruit pericarp contains significant amounts of phenolics (9.39 to 30.16 mg gallic acid equivalents/g fresh weight) and exhibit diverse biological activities. Zhao *et al.* [16] also reported a large amount of polyphenolic compounds with strong antioxidant activity in the pericarp of harvested lychee fruits. According to Zhang *et al.* [25], the free, bound and total phenolic contents were 66.17 to 226.03, 11.18 to 40.54 and 101.51 to 259.18 mg of gallic acid equivalents/100 g, respectively. Higher total phenolics content in this study could be due to the variation of cultivars cultivated in the Eastern region of India and genotype differences in phenolic contents among the litchi varieties, as also reported by Li *et al.* [17].

### 3.2. DPPH Free Radical Scavenging Activity

**Figure 2:**
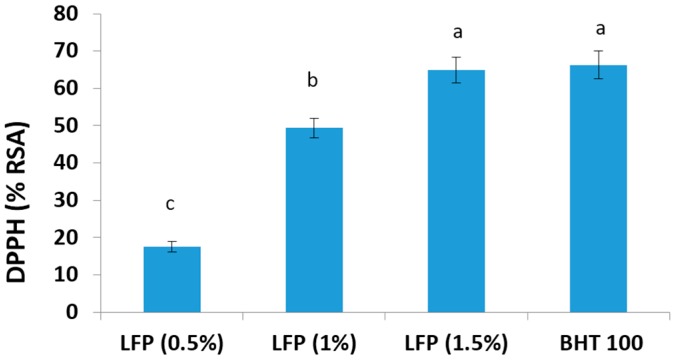
DPPH radical scavenging activity in litchi fruit pericarp extracts (0.5%, 1.0% and 1.5%) and BHT (100 ppm). Mean values bearing different superscripts (a, b and c) differ significantly (*p* < 0.05).

The major beneficial effect of antioxidants when used in biological or food systems include directly quenching of free radicals to terminate the radical chain reaction, chelating transition metals to suppress the initiation of radical formation and stimulating the antioxidative defense enzyme activities [26,27]. The DPPH free radical compound has been widely used to test the free radical scavenging ability of various food samples, and the antioxidant present in the extract neutralizes the DPPH by the transfer of an electron or hydrogen atom [1]. The DPPH radical scavenging activity of litchi fruit pericarp extract was detected and compared to BHT (Figure 2). The radical scavenging activity of litchi fruit pericarp extract possessed excellent antioxidant capacity, and it inhibited the activity of DPPH radicals in a dose-dependent manner. The DPPH radical scavenging activity of 15 mg litchi fruit pericarp powder was comparable to the activity of 100 ppm BHT. Prasad *et al.* [18] reported similar DPPH radical scavenging activity of a litchi fruit pericarp sample obtained by high-pressure extraction. Similarly, higher radical scavenging activity (91.3%) has been reported for anthocyanins present in litchi pericarp [15]. Robards *et al.* [28] reported that there is a linear correlation between radical scavenging activity and polyphenolic content from a wide range of vegetables and fruits. In the case of litchi fruit pericarp extract, a significant correlation was also found between DPPH activities and total phenolic and total flavonoid contents [17]. They also suggested that the phenolics/flavonoids apparently contributed to the antioxidant capacity of litchi fruit pericarp.

### 3.3. Ferric Reducing Antioxidant Power Assay

**Figure 3:**
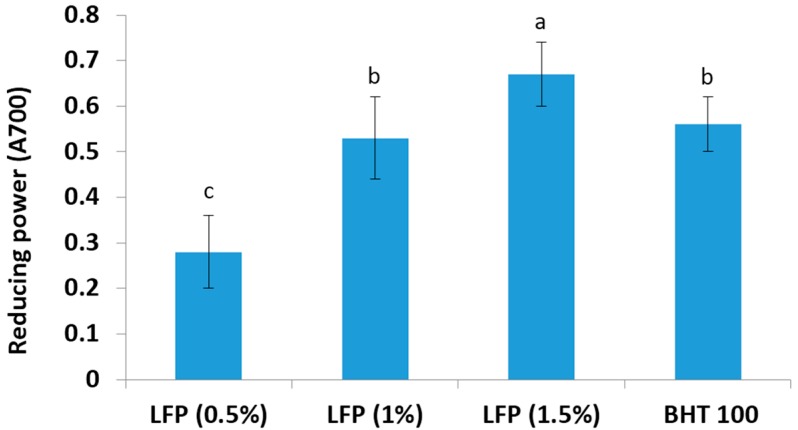
Ferric reducing antioxidant power of litchi fruit pericarp extracts (0.5%, 1.0% and 1.5%) and BHT (100 ppm). Meat values bearing different superscripts (a, b and c) differ significantly (*p* < 0.05).

The reductive capabilities of plant extracts can serve as a significant indicator of their potential antioxidant activities [29]. The potassium ferricyanide reduction method is a widely-used method for evaluating the reducing power of plant polyphenols. In this assay, litchi fruit pericarp extract reduced the iron^3+^/ferricyanide complex to the ferrous form by donating an electron, and it was compared to that of BHT, which is known to be a strong reducing agent. The reducing power of a compound serves as a significant indicator of its potential antioxidant activity [7]. The reducing power of difference concentrations of litchi fruit pericarp extract and BHT is presented in Figure 3. The data obtained in this study revealed that the reducing power of extract from 10 mg LFP powder was comparable with 100 ppm BHT, but 15 mg powder had significantly (*p* < 0.05) higher reducing power. Moreover, higher ferric antioxidant reducing activity at a lower concentration of litchi extract indicates its potential as an antioxidant for food application at the commercial level. Previous studies have also reported that the higher reducing power is associated with more antioxidant activity [7,30].

### 3.4. Total Phenolics, pH and Cooking Yield of Nuggets

The total phenolics content, pH and cooking yield of nuggets with litchi fruit pericarp extract (0%, 1% and 1.5%) and BHT is presented in Table 2. The total phenolics content of sheep meat nuggets prepared with LFP extract was significantly (*p* < 0.05) higher compared to control nuggets, but nuggets with 1.5% LFP extract had comparable total phenolics with 100 ppm BHT nuggets. Similarly, Banerjee *et al.* [10] reported significantly higher total phenolics content in goat meat and nuggets incorporated with BHT than control nuggets. Verma *et al.* [31] also reported that incorporation of guava powder in meat products’ formulation significantly increased the phenolics content of final products than the control. Sheep meat nuggets prepared with 1% and 1.5% LFP extract had comparable pH value with those of control and BHT nuggets, but nuggets with 100 ppm BHT had lower pH value compared to others. Similar lower pH values of meat products with BHT were also reported by other workers [7,32]. However, the addition of LFP extract or BHT did not affect the cooking yield of sheep meat nuggets.

### 3.5. Sensory Analysis

The sensory attributes of different sheep meat nuggets with respect to appearance, flavor, texture, juiciness and overall acceptability are presented in Table 3. The use of BHT (100 ppm) and different concentrations of litchi pericarp extract did not influence the sensory attributes of sheep meat nuggets. The sensory attributes of control, litchi fruit pericarp extract and BHT-treated nuggets were almost similar. Similarly types of studies also report that the addition of kinnow rind powder, pomegranate rind powder and pomegranate seed powder extract do not exert any negative effect in cooked goat meat patties [12,32].

### 3.6. Lipid Oxidation in Sheep Meat Nuggets

**Figure 4:**
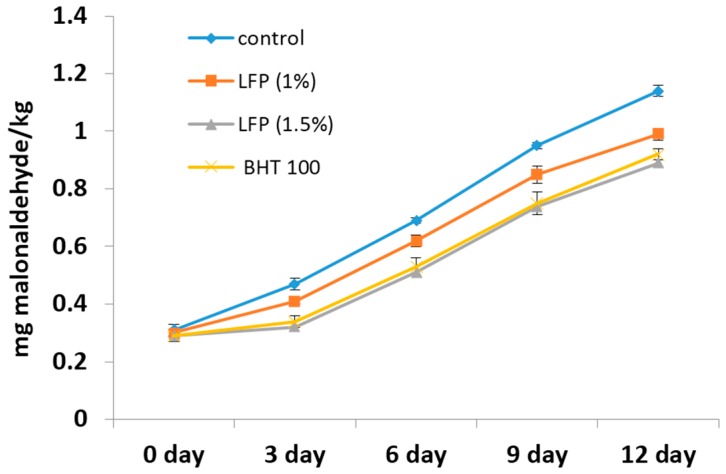
Thiobarbituric acid reactive substance values in sheep meat nuggets with litchi fruit pericarp extracts and BHT during refrigerated storage (4 ± 1 °C).

The determination of the lipid oxidation was done to test the antioxidant effectiveness of litchi fruit pericarp extract as compared to control and BHT-incorporated nuggets under refrigerated storage condition (0, 3, 6, 9 and 12 days) and is presented in Figure 4. The initial concentration of TBARS in the control nuggets, as well as in all of the treated nuggets was between 0.31 ± 0.01 and 0.29 ± 0.01 mg malonaldehyde/kg, and the values were not significantly different. Compared to the control, the TBARS values of nuggets with 1.5% extract and 100 ppm BHT were significantly (*p* < 0.05) lower on the 3rd, 6th, 9th and 12th day of refrigerated storage. TBARS values in control nuggets increased from 0.31 to 1.14 mg malonaldehyde/kg sample, whereas lipid oxidation in nuggets with 1.5% and 100 ppm BHT remained lowest (0.29 to 0.92 mg malonaldehyde/kg sample) during the 12-day storage period. The antioxidant effect of LFP extract in the inhibition of lipid oxidation could be due to the presence of higher phenolics content by inhibiting free radical formation and by the propagation of the free radical chain reaction through the chelation of metals. Prasad *et al.* [18] found that litchi pericarp extract contained high phenolics and flavonoid compounds exhibiting strong antioxidant activity. This may be due to the anti-oxidative activity of phenolic compounds by scavenging free radicals, thereby decelerating lipid oxidation [33,34,35] or metal-chelating activity. Previous studies have also reported that incorporation of plant extracts containing phenolic and flavonoid compounds retarded lipid oxidation in meat products during storage [9,36]. Therefore, litchi pericarp extract (1.5%) could be used effectively to inhibit lipid oxidation in sheep meat nuggets.

## 4. Conclusions

The present findings indicate that litchi fruit pericarp powder is a good source of phenolic compounds having strong free radical scavenging activity and reducing power. The incorporation of pericarp extract in sheep meat nuggets did not have any adverse effect on pH, cooking yield and sensory attributes. Litchi pericarp extract at 1.5% significantly increased the phenolic content in sheep nuggets compared to other treatments and was effective at inhibiting the lipid peroxidation of cooked nuggets similar to the synthetic antioxidant BHT (100 ppm) over a period of 12 days. Being a promising natural antioxidant, litchi fruit pericarp extract could, therefore, be used effectively to improve the product quality, stability and safety of different meat and meat products.

## Figures and Tables

**Table 1:** Sheep meat nuggets’ formulation prepared with litchi fruit pericarp (LFP) extract and butylated hydroxytoluene (BHT).

Ingredients	Control	LFP (1%)	LFP (1.5%)	BHT (100)
Meat (%)	71.1	70.1	69.5	71.1
Salt (%)	1.8	1.8	1.8	1.8
Ice flakes (%)	10	10	10	10
Refined oil (%)	8	8	8	8
Condiments (%)	4	4	4	4
Polyphosphate (%)	0.3	0.3	0.3	0.3
Dry spice mix (%)	1.8	1.8	1.8	1.8
Na nitrite (ppm) (%)	150	150	150	150
Wheat flour (%)	3	3	3	3
LFP extract (%)	0.00	1.00	1.5	-
BHT (100 ppm)	-	-	-	100

Control: no LFP extract; LFP (1%): sheep meat nuggets with 1% LFP extract (equivalent to 10 mg LEP powder); LFP (1.5%): sheep meat nuggets with 1.5% LFP extract (equivalent to 15 mg LEP powder); BHT100: sheep meat nuggets with 100 ppm BHT; Condiments: onion and garlic (4:1). Dry spice mix: aniseed, black pepper, capsicum, caraway seed, cardamom, cinnamon, cloves, coriander powder, cumin seed, turmeric and dried ginger.

**Table 2:** Effect of litchi fruit pericarp extracts and BHT on pH, product yield and total phenolics of sheep meat nuggets.

Measurements	Control	LFP (1%)	LFP (1.5%)	BHT (100)
pH	6.21 ± 0.03	6.20 ± 0.02	6.22 ± 0.02	6.19 ± 0.02
Cooking yield (%)	93.62 ± 0.48	93.29 ± 0.36	94.12 ± 0.43	93.75 ± 0.39
Total phenolics (GAE) mg/g	0.05 ± 0.01 ^c^	0.13 ± 0.01 ^b^	0.17 ± 0.01 ^a^	0.16 ± 0.01 ^a^

Control: no litchi fruit pericarp extract; LFP (1%): sheep meat nuggets with 1% LFP extract; LFP (1.5%): sheep meat nuggets with 1.5% LFP extract; BHT (100): sheep meat nuggets with 100 ppm BHT. Means ± SE bearing different superscripts (^a,^^b^ and ^c^) differ significantly (*p* < 0.05).

**Table 3:** Sensory attributes of sheep meat nuggets incorporated with litchi fruit pericarp extracts and BHT.

Sensory Attributes	Control	LFP (1%)	LFP (1.5%)	BHT (100)
Appearance	7.23 ± 0.05	7.22 ± 0.06	7.18 ± 0.06	7.12 ± 0.04
Flavor	7.06 ± 0.6	7.05 ± 0.08	7.03 ± 0.06	6.94 ± 0.07
Texture	7.15 ± 0.08	7.03 ± 0.07	7.01 ± 0.06	7.02 ± 0.06
Juiciness	7.07 ± 0.07	7.13 ± 0.04	7.15 ± 0.05	7.17 ± 0.05
Overall acceptability	7.18 ± 0.05	7.08 ± 0.06	7.11 ± 0.06	7.05 ± 0.07

Control: no litchi fruit pericarp extract; LFP (1%): sheep meat nuggets with 1% LFP extract; LFP (1.5%): sheep meat nuggets with 1.5% LFP extract; BHT100: sheep meat nuggets with 100 ppm BHT. Attributes were measured on an 8-point scale with 8 = extremely desirable and 1 = extremely poor.
